# Multifactor Assessment of Non-Antimicrobial Soap Performance

**DOI:** 10.1093/ofid/ofz151

**Published:** 2019-03-20

**Authors:** James Bingham, Todd J Cartner, Patricia A Mays Suko, Rachel A Leslie

**Affiliations:** 1 Research and Development, GOJO Industries, Inc., Akron, Ohio; 2 Bioscience Laboratories, Inc., Bozeman, Montana

**Keywords:** hand hygiene, handwash, healthcare personnel, infection prevention, infection control, transmission

## Abstract

A non-antimicrobial soap was benchmarked against 2 reference soaps for microbial removal and skin compatibility, key factors in soap effectiveness and usage. The non-antimicrobial test soap removed more *Staphylococcus aureus* (*P* = .024) when applied to nonwetted hands and showed no difference in skin barrier function compared with the reference soaps (*P* = .736).

The clinical effectiveness of hand hygiene programs is dependent on the complex relationship between multiple factors including hand hygiene products, education programs, hand hygiene monitoring and feedback practices, and an institutional safety climate [1, 2]. For hand hygiene products, the efficacy profile—killing or removing microorganisms—and acceptance by healthcare personnel (HCP)—skin compatibility and user experience—are factors that influence their performance and usage [1, 2]. Both non-antimicrobial and antimicrobial soaps are acceptable for use in healthcare settings per the Centers for Disease Control and Prevention (CDC) Hand Hygiene guideline [1]; however, the safety and efficacy of many antimicrobials is currently under scrutiny by the US Food and Drug Administration [3]. Therefore, there is a growing interest and need for non-antimicrobial soaps that meet healthcare needs in microbial removal, skin compatibility, and user acceptance.

The microbial removal by non-antimicrobial soaps has been reported, but broad conclusions are difficult to make due to methodological, organism, and soap variation between studies [4]. Skin compatibility is a significant issue with soaps because frequent use can cause irritation and dryness of HCP hands, which influences their acceptance and use [2, 5]. Studies have shown that damaged skin can lead to chronic skin conditions, such as dermatitis [6, 7]. User experience also has a significant impact on product use [2]; however, there is little published data comparing products or the key aspects that drive user experiences. Although the published studies evaluating the microbial removal and impacts on skin condition by non-antimicrobial soap provide foundational evidence of the behavior of soap, they are not actionable regarding product selection for healthcare facilities. Furthermore, there are no published reports of controlled experiments comparing non-antimicrobial soaps on multiple factors that impact product performance and usage. The purpose of this study was to benchmark a new foaming soap against 2 reference soaps with standardized industry methods under 2 application conditions for key characteristics that influence performance and usage—microbial removal and skin compatibility.

## METHODS

### Test Products

Three commercially available non-antimicrobial soaps manufactured by GOJO Industries were tested in this study: (1) a base soap—Reference Soap A (comprised of water, sodium laureth sulfate, citric acid, cocamidopropyl betaine, disodium cocoamphodiacetate, glycerin, polyethylene glycol (PEG)-7 glyceryl cocoate, polyquaternium-39, methylchloroisothiazolinone, methylisothiazolinone; (2) Reference Soap B, a modification of the base soap for skin health through increased glycerin and additions of PEG-8 and sodium pyroglutamic acid (PCA); and (3) Test Soap, a modification of the base soap for skin health and microbe removal through increased glycerin, addition of PEG-8, sodium PCA, ethyl alcohol, and trisodium ethylenediamine-*N,N*′-disuccinic acid and removal of methylchloroisothiazolinone and methylisothiazolinone.

### Microbial Removal Study

Test Soap and Reference Soap A were evaluated for the removal of *Staphylococcus aureus* ATCC 6538, a relevant healthcare pathogen, in 2 randomized, crossover studies using ASTM E2755-15 [8], modified to include a rinse step for evaluation of soap. Each study was approved by an Institutional Review Board (IRB): the first study by Gallatin Institutional Review Board (Bozeman, MT) and the second study by Advarra IRB (Columbia, MD). Written informed consent was obtained from all participants. In the first study, 12 participants applied 1.8 mL of either soap, equivalent to 2 actuations from a dispenser, to nonwetted hands, lathered for 30 seconds, and rinsed for 10 seconds. In the second study, 10 participants applied 1.8 mL of either soap to prewetted hands and followed the same wash procedure. Between product applications, hands were decontaminated by washing with a non-antimicrobial soap and a 70% ethanol rinse. A minimum 1-hour rest period was required for participants before testing the other soap. Statistical comparison between test products was made using a paired *t* test, α = 0.05.

### Skin Health Study

Test Soap and Reference Soap B were evaluated for skin compatibility using a Forearm Controlled Application Test (FCAT) [9]. Eight participants signed an informed consent before testing and ceased at-home forearm skin cleansing and moisturizing for the duration of the study. Each of the participant’s forearms were divided into 4 test sites and assigned treatments according to a balanced Latin Square Block Design. Fifty microliters of soap was applied to prewetted and nonwetted skin, rubbed for 10 seconds, rinsed for 10 seconds, and patted dry with a paper towel, mimicking a typical soap dose and handwash process. A total of 48 washes were administered over 4 days (12 washes/day). Skin barrier function was assessed by measuring transepidermal water loss (TEWL) (AquaFlux AF 200; BioX Systems Limited) following European Group for Efficacy Measurements on Cosmetics and Other Topical Products (EEMCO) guidelines [10] before treatment (baseline) and on the fifth day after the 48 treatments (final). The mean difference (baseline compared with final) in TEWL was calculated and statistical analysis was conducted using an analysis of variance General Linear Model, α = 0.05.

## RESULTS

In the first microbial removal study, subjects had an average *Saureus* baseline recovery of 8.39 (±0.23 standard deviation [SD]) log10 colony-forming units (CFU)/hand. After applying soap to nonwetted hands, the Test Soap achieved a 1.46 (±0.36 SD) log10 reduction, which was statistically greater than the 1.12 (±0.32 SD) log10 reduction achieved by Reference Soap A (*P* = .024). In the second study, participants had an average baseline recovery of 8.80 (±0.32 SD) log10 CFU/hand. After soap application to prewetted skin, there was not a statistical difference in the log10 reduction of *S aureus* between the Test Soap (1.07 ± 0.19 SD) and Reference Soap A (0.97 ± 0.33 SD) (*P* = .356).

In the skin compatibility study, comparison between soap application technique—prewetted and nonwetted skin—did not result in a statistical difference in barrier function for either soap (Test Soap, *P* = .433 or Reference Soap B, *P* = 1.000) (Table 1). There was also no statistical difference between the Test Soap and Reference Soap B (prewetted, *P* = .998 or nonwetted, *P* = .736). Compared to controls when applied to nonwetted skin, the Test Soap showed a statistical difference (nonwetted skin, *P* = .036; prewetted, *P* = .0001) in skin barrier function versus 8% sodium lauryl sulfate applied to prewetted skin (positive control) and no statistical difference versus untreated skin (negative control) (nonwetted, *P* = .189; prewetted, *P* = .984).

**Table 1.** Change in Skin Barrier Integrity From Baseline After 48 Handwashes Per Forearm-Controlled Application Test

	Change in TEWL (g/hour m^2^) (Lower Scores Indicate Greater Skin Integrity)		
Treatment	Prewetted Skin Application (±SD)	Nonwetted Skin Application (±SD)	Change in TEWL: Prewetted vs Nonwetted Skin
Test soap	9.86 ± 7.57	13.81 ± 10.21	3.95 *P* = .433
Reference soap B	11.02 ± 10.15	11.81 ± 8.45	0.79 *P* = 1.000
Positive control (8% sodium lauryl sulfate)	24.87 ± 17.87	-	-
Negative control (untreated skin)	-	7.00 ± 5.76	-

Abbreviations: SD, standard deviation; TEWL, transepidermal water loss.

## Discussion

This is the first study to characterize a non-antimicrobial soap on multiple factors that drive performance and usage—microbial removal and skin compatibility. To reduce the transmission of pathogens soaps must reduce, through removal or biocidal action, microbial hand contamination. It was confirmed through in vitro suspension testing based on ASTM E2783 that the non-antimicrobial soaps did not have rapid biocidal activity. The Test Soap and Reference Soap A demonstrated <1 log10 reduction of *S aureus* in 15 seconds (data not shown). The Test Soap removed numerically more bacteria than the Reference Soap A when applied to prewetted skin, but this was not statistically significant. When applied to nonwetted skin, the soaps tested demonstrated significantly different microbial removal results. Because it has been reported that approximately half of users apply soaps to nonwetted skin [11], the difference in microbial removal could be meaningful for a large proportion of hand hygiene events. These results suggest that hand prewetting practices in healthcare should be further understood, and a broader set of non-antimicrobial soaps should be evaluated for microbial removal.

Skin compatibility plays an important role in product usage and, at times, may be the dominant factor. The FCAT study demonstrated no significant differences in skin barrier function between the Test Soap and Reference Soap B, a soap marketed for its mildness. In addition, there was no significant difference between the Test Soap and untreated skin, which is the strongest indication for mildness of the Test Soap. There was no significant difference in skin health when the Test Soap was applied to prewetted and nonwetted skin, demonstrating that the Test Soap can be applied to nonwetted hands. The advent of foam soaps, which do not need water to lather, challenges the need to prewet hands before handwashing as recommended in the CDC Hand Hygiene guideline [1].

In this study, the Test Product was only compared with 1 reference product for each experiment. Additional studies should evaluate more formulations to identify the range of performance of commercially available non-antimicrobial soaps. This study outlines the multidimensional evaluation of hand hygiene products that must be performed to adequately inform product selections due to the complicated relationship between microbial removal, skin compatibility, and HCP acceptance and usage. Future multifactor studies should evaluate hand hygiene products under “real-world” test conditions including application times and doses that reflect actual clinical practice.

Clinical effectiveness of hand hygiene products is dependent on their microbial reduction and HCP usage practices. As non-antimicrobial soaps become more prevalent in healthcare settings, controlled, standardized, and realistic evaluations of microbial removal and product acceptability—including skin compatibility and user experience—are needed to predict their effectiveness.
